# Nutrition and sarcopenia: Current knowledge domain and emerging trends

**DOI:** 10.3389/fmed.2022.968814

**Published:** 2022-10-26

**Authors:** Huanhuan Huang, Zhiyu Chen, Lijuan Chen, Songmei Cao, Dingqun Bai, Qian Xiao, Mingzhao Xiao, Qinghua Zhao

**Affiliations:** ^1^Department of Nursing, The First Affiliated Hospital of Chongqing Medical University, Chongqing, China; ^2^Department of Orthopedic, The First Affiliated Hospital of Chongqing Medical University, Chongqing, China; ^3^Department of Nursing, The Affiliated Hospital of Jiangsu University, Zhenjiang, Jiangsu, China; ^4^Department of Rehabilitation Medicine, The First Affiliated Hospital of Chongqing Medical University, Chongqing, China; ^5^Department of Geriatric, The First Affiliated Hospital of Chongqing Medical University, Chongqing, China; ^6^Department of Urology, The First Affiliated Hospital of Chongqing Medical University, Chongqing, China

**Keywords:** nutrition, sarcopenia, VOSviewer, co-words, bibliometric analysis

## Abstract

**Objective:**

Non-pharmacological management like nutrient supplements has shown positive impacts on muscle mass and strength, which has burgeoned clinical and research interest internationally. The aim of this study was to analyze the current knowledge domain and emerging trends of nutrition-related research in sarcopenia and provide implications for future research and strategies to prevent or manage sarcopenia in the context of aging societies.

**Materials and methods:**

Nutrition- and sarcopenia-related research were obtained from the Web of Science Core Collection (WoSCC) database from its inception to April 1, 2022. Performance analysis, science mapping, and thematic clustering were performed by using the software VOSviewer and R package “bibliometrix.” Bibliometric analysis (BA) guideline was applied in this study.

**Results:**

A total of 8,110 publications were extracted and only 7,510 (92.60%) were selected for final analysis. The production trend in nutrition and sarcopenia research was promising, and 1,357 journals, 107 countries, 6,668 institutions, and 31,289 authors were identified in this field till 2021. Stable cooperation networks have formed in the field, but they are mostly divided by region and research topics. Health and sarcopenia, metabolism and nutrition, nutrition and exercise, body compositions, and physical performance were the main search themes.

**Conclusions:**

This study provides health providers and scholars mapped out a comprehensive basic knowledge structure in the research in the field of nutrition and sarcopenia over the past 30 years. This study could help them quickly grasp research hotspots and choose future research projects.

## Introduction

People worldwide are living longer. According to World Health Organization (WHO), people aged 65 and over would nearly account for 17% of the population by 2050 (1). Aging is always along with a series of physiological and psychological changes, among them, the most conserved hallmarks are the decline of functional capabilities, which strew great challenges to health. Sarcopenia is an age-related disease that is defined as a decrease in muscle quantity and quality, as well as physical performance (2). Sarcopenia and frailty are closely related and there is a diagnostic overlap between sarcopenia and frailty (3, 4), especially in the concept of nutritional frailty (5). It is estimated that the global prevalence of sarcopenia is ranged from 3.3 to 17.5% depending on various diagnostic criteria and assessment tools (6, 7). Compared with the individuals without sarcopenia, those having low grip strength, lean mass, strength, power, or physical function have more grave physiological and clinical consequences (8) and have high risks of mobility, fall, and disability (9). Although some research suggests that hormonal changes (10), oxidative stress (11, 12), and mitochondrial dysfunction (13) may play a great role in the development of sarcopenia, the pathophysiology of the disease is complex and not yet fully elucidated (4). Accordingly, understanding more about sarcopenia’s risk factors and its coping strategies is of great interest.

Non-pharmacological management like nutrient supplements has shown positive impacts on muscle mass and strength, which has burgeoned clinical and research interest internationally. Notably, due to the decline of physical exercise and a lower need for energy intake, as well as the difficulty in the assessment of nutritional status in frailty phenotype (3), community-dwelling older adults have a high risk of being at nutritional or becoming malnourished (14), and a large body of evidence has linked malnutrition with the negative health effects of the old on muscles (15, 16). Based on the International Clinical Practice Guidelines for Sarcopenia (ICFSR) (17), preserving or restoring adequate nutritional status is of great significance for the prevention and optimal management of sarcopenia. Studies have proved that prevent of general malnutrition or micronutrient deficiencies has some potential to promote the physical performance (18–20). Moreover, serval randomized controlled trials conducted in older adults with sarcopenia showed that nutrition intervention was not only feasible (21) but also safe (22). Given that nutrition may influence the development of sarcopenia, this topic deserves further discussion. However, despite the approximately 30-year history of sarcopenia research, a detailed quantitative analysis of the existing research has not been undertaken to elucidate the body of evidence on the nutrition research in the sarcopenia field. Thus, we aimed to analyze the current knowledge domain and emerging trends of the evidence that associates nutrition with muscle quality and quantity, and physical performance. Our study helps to bridge this gap and provide implications for future research and strategies to prevent or manage sarcopenia for clinical health providers, and attribute to gain a one-stop overview for the readers in the sarcopenia literature.

## Materials and methods

### Study design

**FIGURE 1:**
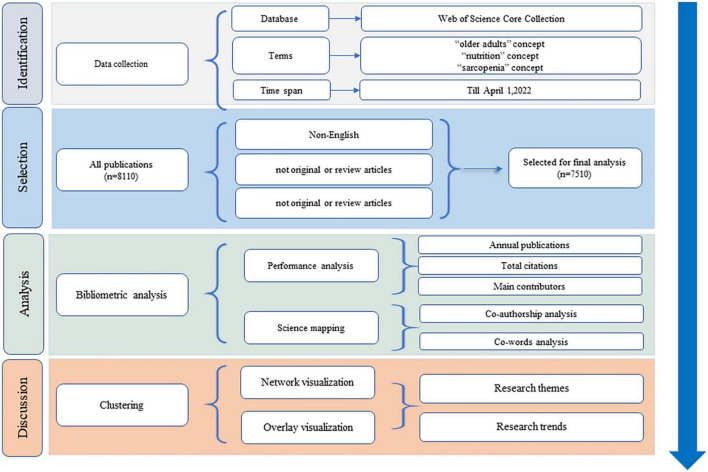
Flow chart of the research framework.

As an important carrier of scientific research, academic publications could clearly reflect the basic knowledge domain and emerging trends of a certain discipline (23). Bibliometric analysis (BA) is a quantitative and comprehensive method associated with academic publications (24). This method could reveal the current knowledge domain through performance analysis with publication-related and citation-related metrics (25). In addition, mathematics, statistics, and philology methods are used to identify emerging trends through science mapping with citation, co-citation, or co-word analysis (26). To date, BA has been widely used to analyze the progress of research fields and to predict the development of disciplines due to its objectivity, quantitative, and macro characteristics compared to systematic or meta-analysis (27). Thus, BA and its guideline (28) were applied in this study, as shown in Figure 1. This study was not reviewed by the ethics committee for neither patients nor members of the public were involved.

### Data source

Different databases (e.g., Scopus and Web of Science) have their own format of bibliometric data. BA guideline suggested that choose one appropriate database to mitigate the need of consolidation, as minimizing unnecessary action items can help to prevent potential human errors (28). The Web of Science Core Collection (WoSCC) of Clarivate Analytics including Science Citation Index Expanded, Social Sciences Index, and Arts & Humanities Citation Index, is regarded as one of the most complete and reliable databases for BA (29), which can retrieve the references of publications and track the latest citation (30). Therefore, all data used in this study were retrieved from the WoSCC.

### Search strategy

On April 1, 2022, the WOSCC was searched using topic words and keywords plus. The search terms were “nutrition intake,” “dietary supplements,” “sarcopenia,” “muscle strength,” “muscle function,” and “muscle mass.” The strategy of Behnaz et al. (31) and Dongliang et al. (32) were mainly referenced during the process of string construction. After selecting all the relevant search terms and their combinations, Boolean operators and a general review were performed. English language, incorporated animal and human studies were acceptable, and no restrictions on the dates of publications.

### Data collection

**FIGURE 2:**
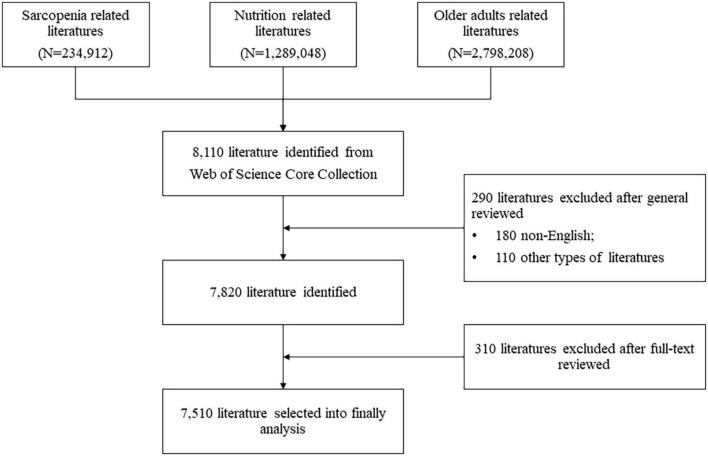
Flow chart of the data collection procedure.

Two authors manually and independently evaluated the title and abstract of the selected publications. Publications without abstracts were full-text reviewed. The third author verified the consistency of the results. Finally, a total of 8,110 publications were identified from WoSCC and only 7,510 were selected for final analysis, 180 non-English documents, 110 were not original or review articles were excluded after general reviewing, and 310 were excluded for not associated with the topic of nutrition or sarcopenia after evaluated the title and abstract. Figure 2 showed the flow chart of the data collection procedure.

### Bibliometric analysis

Full records and cited references of the publications were downloaded in txt format, and then input to the VOSviewer version 1.6.15 (Leiden University, The Netherlands) and R package “bibliometrix” version 4.1.2, thesaurus file was used for data cleaning. The steps of analysis were as follows: (A) performance analysis was conducted to explore the current knowledge domain, which includes the annual publications, total citations, and main contributors, such as topics, journals, and core authors. Core authors was defined by Price’s Law (33), that is, $m_{p}=0.749\,\sqrt{n_{p\,m\,a\,x}}$ in which *n*_*pmax*_ is the output of the most prolific authors, *m*_*p*_ is the minimum number of output of core authors in the selected period. (B) Science mapping was conducted to analysis the emerging trends on science collaboration and research hotpots, which including co-authorship and co-words. (C) The thematic clustering was used to identify the research themes and evolution features according to Zipf’s Law (34) by network and overlay visualizations.

## Results

### Annual publications and total citations

**FIGURE 3:**
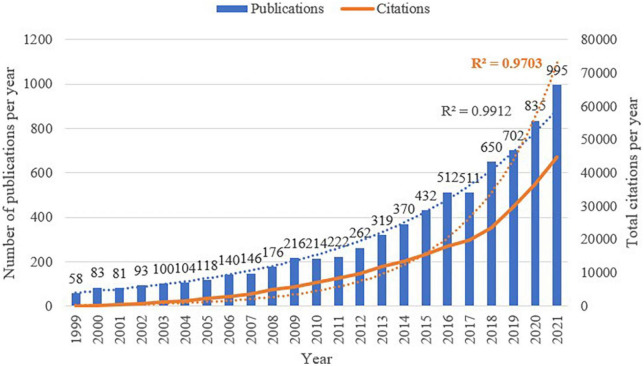
Annual publications and total citations number from 1999 to 2021.

A total of 7,510 publications were selected for final analysis. As shown in Figure 3, though the publications have dropped slightly in some years, annual publications related to nutrition in the sarcopenia field quickly increase from 58 in 1999 to 995 in 2021, showing a clear trend for growth. The correlation between the number of publications and the year was significant (*R*^2^ = 0.9912). The total citations also showed a similar upward trend, with a correlation coefficient of 0.9703, the number jumped from 30 in 1999 to 44,585 in 2021. Convincingly, the publication and citation in the filed of nutrition- and sarcopenia-related research would reach a new milestone at 2022.

### Main contributors

#### Topics and journals

**FIGURE 4:**
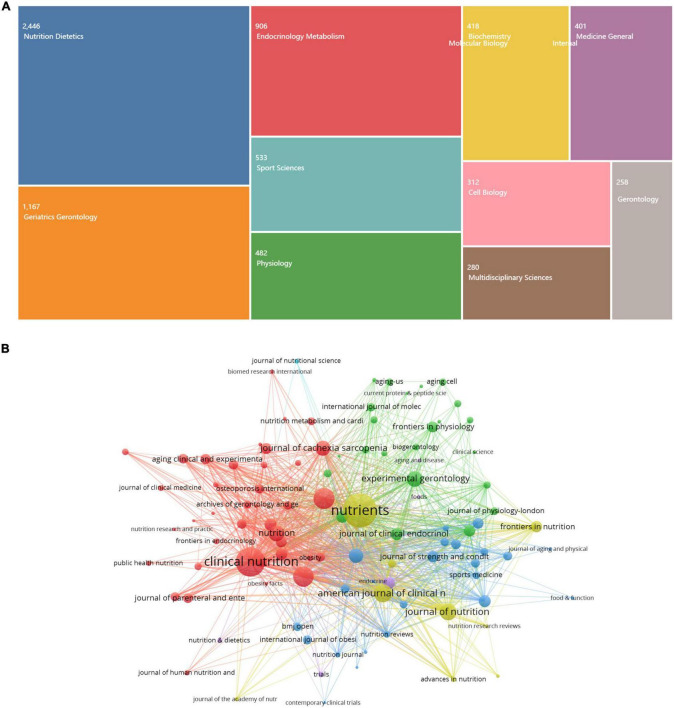
The main categories and journals in the nutrition-related sarcopenia research. **(A)** The top 10 journal citation reports (JCR) category of topic themes. **(B)** The top 100 most productive journals.

Figure 4A showed the top 10 category in the nutrition- and sarcopenia-related research, among which the main topics were nutrition dietetics, followed by geriatrics gerontology, endocrinology metabolism, and sport science, covering a wide range of academic disciplines, indicating this filed was a multi-disciplinary work.

Journals are an important carrier of scientific research. Analyzing the source journals helps understand the research field it belongs to, and for other scholars to evaluate the development potential of this hotspot. Figure 4B showed the top 100 productive journals, in which color bubbles represent the number of publications in each journal, and lines indicate cooperation between two journals. Among which, *Nutrients* (406, 5.41%) from MDPI press ranked first in production, followed by *Clinical Nutrition* (319,4.25%), and *PLoS One* (174, 2.32%). Table 1 lists the ten journals with the largest number of nutrition-related research in sarcopenia field and their academic influence index. Almost all belonged to Q1 in JCR, and with an average influence factor of 5.92. Besides, *the Journal of Cachexia Sarcopenia and Muscle* have highest impact factor with 12.91.

**TABLE 1:** Top 10 productive journals in the nutrition-related sarcopenia research.

Rank	Source	Documents	Counts	Citations	IF (2021)	JCR	Region
1	Nutrients	406	5.41%	5,869	5.719	Q1	Switzerland
2	Clinical nutrition	319	4.25%	14,651	7.325	Q1	Scotland
3	PLoS One	174	2.32%	4,260	3.24	Q2	USA
4	Journal of nutrition health and aging	171	2.28%	4,870	4.075	Q2	France
5	American journal of clinical nutrition	134	1.78%	12,980	7.047	Q1	USA
6	Journal of nutrition	117	1.56%	5,250	4.798	Q1	USA
7	Nutrition	104	1.38%	2,588	4.008	Q2	USA
8	Experimental gerontology	102	1.36%	2,630	4.032	Q2	England
9	Journal of cachexia sarcopenia and muscle	98	1.30%	2,683	12.91	Q1	Germany
10	Journals of gerontology series A-biological sciences and medical Science	92	1.23%	5,579	6.053	Q1	USA

IF, impact factor; JCR, journal citation reports; the US, The United States.

#### Countries, institutions, and authors

After reviewing the contributors, a total of 107 countries, 6,668 institutions, and 31,289 authors were identified in the nutrition- and sarcopenia-related research field. Among those, the most productive country was the USA (2,284, 30.41%), followed by the United Kingdom (UK), Japan, Italy, and Canada, counting for 10.48, 8.08, 7.79, and 7.18% of the total publications, respectively. And Maastricht University (*n* = 138) and McMaster University (*n* = 104) were the leading institutions in terms of productivity.

**FIGURE 5:**
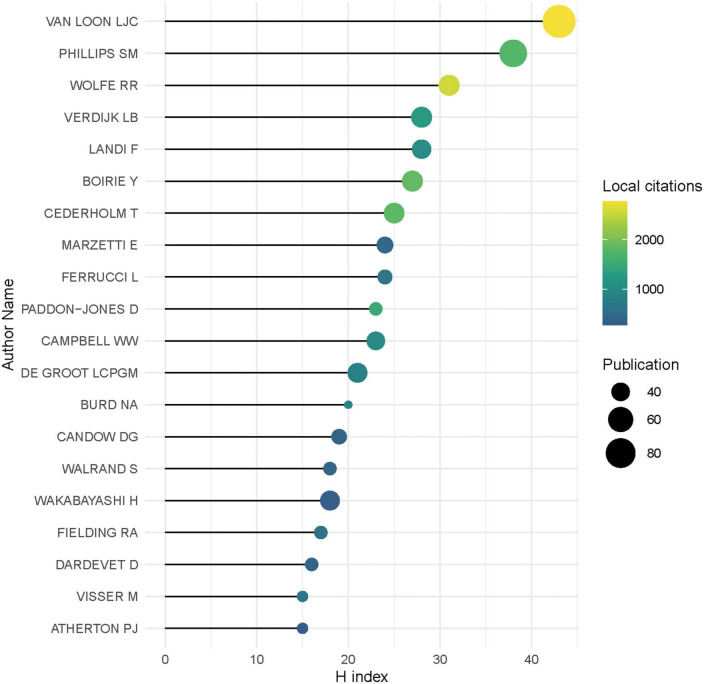
The top 20 most prolific authors and their academic impact.

Besides, Professor van Loon L.J.C. from Maastricht University Medical Centre was found to be the most productive with 96 publications. And according to Price’s Law, core authors should publish at least 7.34 publications, that is, those who publish 8 articles or more could be identified as core authors. In other words, there were 437 core authors in the field of nutrition-related research in sarcopenia. Figure 5 showed the top 20 most prolific authors and their academic impact, in which Professor van Loon L.J.C with the highest H index of 43 and most local citations as well.

### Co-authorship analysis

**FIGURE 6:**
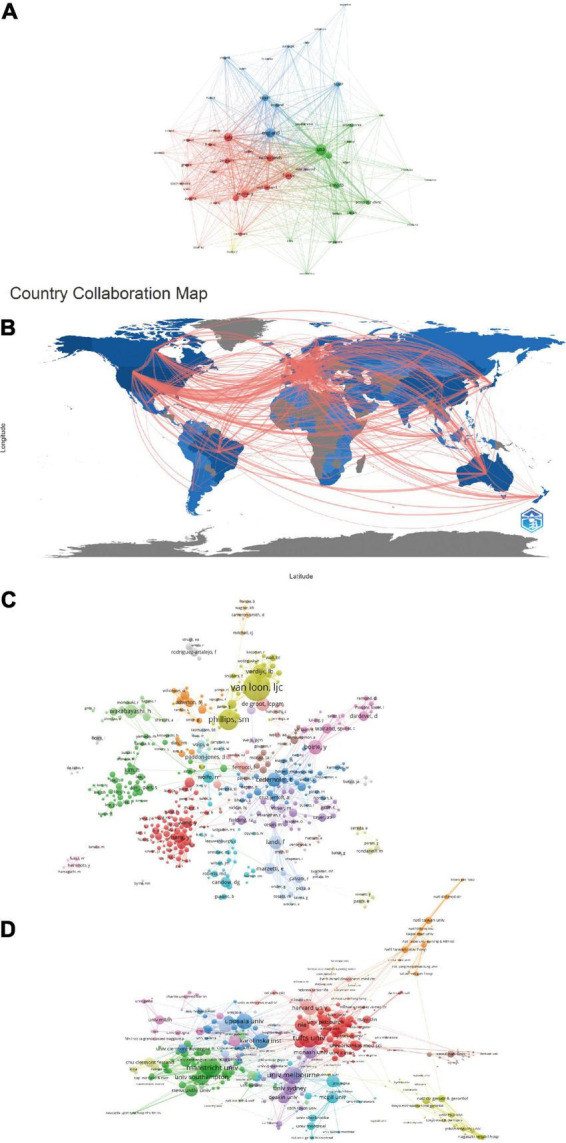
Co-authorship map among **(A)** productive countries, **(B)** countries, **(C)** authors, and **(D)** institutions on nutrition-related research in sarcopenia.

Scientific cooperation occurs when scholars in relevant research fields work together to innovate scientific knowledge (35). Figure 6 showed the collaboration among countries among (A) productive countries, (B) countries, (C) authors, and (D) institutions on nutrition-related research in sarcopenia, in which each circle and label forms an element, the size of the element depends on the number of publications of the contributor, the strength of the element depends on the frequency of collaboration between two contributors, the color of the element represents the cluster of the research topic to which it belongs. In general, Figure 6 implied that compared with a country’s collaboration, institutions and authors’ collaboration provides a measure to examine interactions between agencies at a more granular level (36, 37).

### Co-words analysis

One of the main functions of keywords is to provide core information about the article. At the same time, keywords are important for bibliometric analyses of the knowledge structure in an academic field, for similar articles are prone to adapt to neighbor keywords (38). Thus, research mapping was explored by investigating the co-occurrence of all keywords in the titles and abstracts of 7,510 documents, and clustering was applied to identify the emerging trend.

**FIGURE 7:**
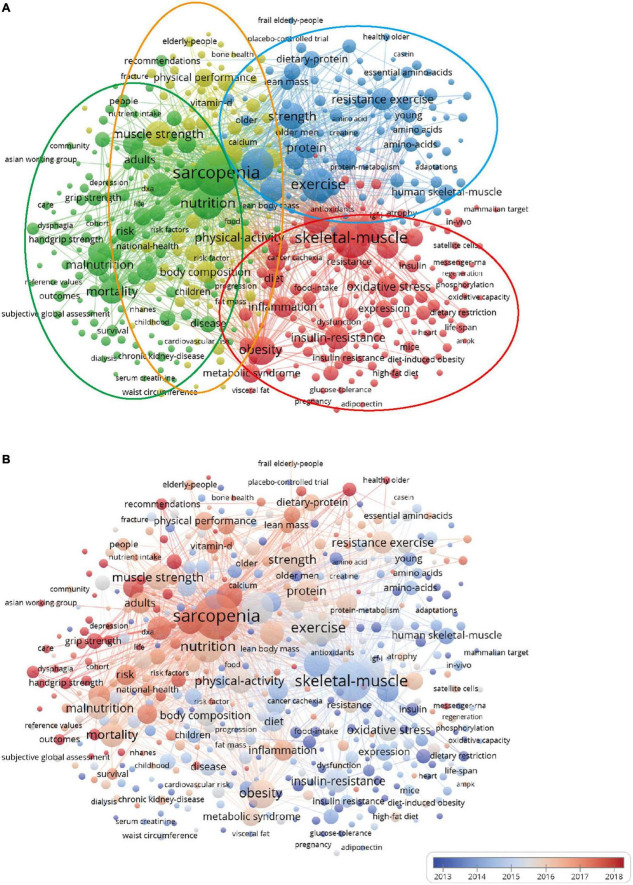
The co-words analysis of the top 500 keywords. **(A)** Network visualization; **(B)** overlay visualization.

Figure 7 was a bubble chart of the co-words network of top 500 keywords. Figure 7A showed the network visualization, in which keywords were divided into four categories (red, blue, yellow, and green), indicating the four mainstream research topics in this filed: the first one in green centered on health and sarcopenia, associated with malnutrition, risk, and mortality; the second in red on metabolism and nutrition, associated with oxidative stress, insulin resistance, and obesity; the third in blue on nutrition and exercise, mainly associated with resistance exercise; and lastly in yellow, on body compositions and physical performance, associated with skeletal strength, vitamin D, calcium, magnesium (39, 40). Figure 7B showed the overlay visualization and colors represented the time of evolution, in which clusters of green and yellow were the hotpot and future research trends in the nutrition- and sarcopenia-related field.

## Discussion

Bibliometric analysis method offers a one-stop overview to identify evolutionary nuances in different fields and capture emerging trends (41). In this study, we used BA quantitative method to analyze the development of nutrition- and sarcopenia-related research at a global level, which would help sarcopenia and nutrition researchers gain a more comprehensive understanding of the current state of this topic and thus guide the direction of future research. Compared with other BA studies that only focus on single journals (42, 43) or are restricted to a specific time (32, 44), and unlike Meta-analysis or systematic literature review (45, 46) that aim to summarize and synthesize the findings of small literature on a specific research topic or field, our study provides scholars with a comprehensive basic knowledge structure and research trend.

We found that the nutrition- and sarcopenia-related field has shown a growing trend over the past 30 years. A similar trend was also observed in a few BA studies on sarcopenia along, which found the publication number rise dramatically between 2008 and 2010 (44, 47). One of the reasons is the incrassating recognition of the definition and diagnosis of sarcopenia. Actually, sarcopenia has a relatively short history, it was not until 1989 that Irwin Rosenberg proposed the term “sarcopenia” (48). The European Working Group on Sarcopenia in Older People (EWGSOP) was established in 2009 and developed the first practical clinical definition and consensus diagnostic criteria for this disease in 2010 (49) and, therefore, increased research interests in this field.

Our BA results also demonstrated that nutrition-related sarcopenia studies have become a research hotspot in not only nutrition dietetics and geriatrics gerontology but also multiple disciplines, like endocrinology metabolism and sports science. In fact, up to 108 Web of Science categories were identified in this field. The potential explanation is that skeletal muscle is not only affecting the energy and protein metabolism throughout the body, but also plays an essential role in body movement and daily actions such as chewing, swallowing, and breathing (50). Overall, we emphasize the important role of close multidisciplinary collaboration in the prevention and treatment of sarcopenia. In addition, studies in this field are published in a wide range of journals covering many specialties, among which a large number of publications from *Nutrients* were impressive. Notably, *Nutrients* also own high citations in this field, which means the findings reported have been useful to other researchers for initiating, performing, or interpreting their own research.

We focused on the main contributors with productive performance in this field, the USA stands out from all countries as hegemony in the production of knowledge, followed by the UK, Japan, Italy, and Canada, and this may be due to those mentioned countries stepped into aging at the earliest. We also identified the most productive academic institutions in the relevant studies, and the result is similar to the finding on the contribution of countries. Interestingly, we recognized the core authors in the nutrition-related sarcopenia studies for the first time according to Price’s Law, such as Loon L.J.C. We also ranked who with a comprehensive index, like total publications, local citations, and H index, which would provide more insights on the most influential author. It is important to note, however, that we only included the studies published in English, thus some individuals’ productivity may be underestimated.

Moreover, from the perspective of macro-geographical distribution, the results of co-authorship analysis suggest that several relatively stable cooperation networks have been formed in the field of nutrition -related research in sarcopenia, but they are mostly divided by region and research topics. Therefore, cross-institutional and cross-border collaboration between main contributors needs to be further strengthened.

From a methodological standpoint, our study allows us to identify themes using co-word analysis. Compared with other publications, the results refreshed the ideas of some highly cited articles relating nutrition to sarcopenia (51–53). On the other hand, our study provided a larger database size for analysis, moreover, we not only analyzed the relationship between selected publications and clustered those with a quantitative method (54, 55), but also revealed the intellectual bases and research hotspots in this field.

Focusing on the current knowledge domain and emerging trends, nutrition-related sarcopenia studies would continue to conduct more in-depth large sample study and mechanism research in hotspots and fronts field. First, in terms of health and sarcopenia, it is expected more raw, longitudinal data and publicly available secondary data will be applied to explore the negative results and risk factors of malnutrition among the sarcopenia population. Second, is the discussion of the potential metabolism pathway of nutrition. As the major organ of insulin-induced glucose metabolism in the body, the loss of quantity and quality of skeletal muscle is associated with a complex of pathologies (56). Thus, those findings could provide an insight into the molecular pathogenesis of sarcopenia and a potential target for new nutrition interventions. Third, the combined intervention and mutual effect among nutrition and exercise, in which randomized controlled trial designs were overwhelming and promising. Last, is the research on body compositions and physical performance. It is relatively broad and has an obvious overlap with the other three clusters. However, the overlay visualization emphasizes its frontier, indicating that this theme is playing a vital role in the field, and the supplements and consumption of Vitamin D, and minerals (57) (magnesium, selenium, iron, zinc et al.) are possible directions for breakthroughs in the future.

## Limitations

Although efforts have been made to retrieve various publications relevant to nutrition-related sarcopenia studies, as a novel disease, the terms included in this study and the period may not be extensive enough. In addition, this study only searched in one database and restricted the language to English, which may limit the inclusion of relevant studies, especially those published in other languages. Last but not least, the practical implementation of citation analyses and co-words requires appreciable expertise. Thus, in subsequent studies, we will further optimize data sources and data filtering to improve the quality of overall data analysis and prediction, and synthetic knowledge synthesis would be applied to formally define themes, categories, and concepts.

## Conclusion

In this study, a comprehensive BA of the nutrition-related sarcopenia study was performed using WoSCC data. Different visualization methods were used to interactively explore and understand specific data sets. Based on the above results and discussions, some valuable results for the nutrition and sarcopenia study were obtained, including the knowledge domain and emerging trends. In conclusion, the role of nutrition in sarcopenia has received growing research attention, with the USA having the largest number of publications. This study has also identified the main contributors involved in this research globally, and at the same time, it is reasonable to believe that the collaboration between different contributors will be strengthened when the core group is formally established among countries, institutions, and core authors. In addition, the themes of nutrition- and sarcopenia-related research currently could be divided into four categories, more in-depth large sample studies and mechanism research in hotspots and fronts field are needed.

## Data availability statement

The raw data that support the findings of this study are available from the corresponding author upon reasonable request.

## Author contributions

HHH: conceptualization and writing – original draft. ZYC: formal analysis and review and editing. QHZ: funding acquisition. HHH and LJC: methodology. SMC and QHZ: project administration. DQB and QX: supervision. MZX and QHZ: validation. All authors have read and agreed to the published version of the manuscript.
